# Supplementary material for: Factors associated with measles vaccine immunogenicity in children at University Teaching Hospitals, Lusaka, Zambia
Source: PLOS Glob Public Health. 2025 Apr 23;5(4):e0003954. doi: 10.1371/journal.pgph.0003954 (PMC12017566; doi:10.1371/journal.pgph.0003954)
Supplement: Attachment — Submitted filename: Responses to Reviewer.docx [file pgph.0003954.s004.docx]

**Rebuttal Letter**

**Manuscript ID:** PGPH-D-24-02312

**Title:** Factors Associated with Measles Vaccine Immunogenicity in Children at University Teaching Hospitals, Lusaka, Zambia

**Journal:** PLOS Global Public Health

Dear Editor,

Thank you for the opportunity to revise and improve our manuscript. We have addressed each of the points raised and believe that these revisions enhance the clarity and depth of the study. Our responses to each comment are outlined below.

**Editor Comments and Author Responses**

**Abstract Methods Alignment with Research Question**

**Comment:** Could you make the abstract methods more in line with your specific research question?
**Response:** We have revised the methods section of the abstract to align more closely with our specific research question, which examines the relationship between age, HIV status, breastfeeding history, and socio-demographic factors on measles immunogenicity. The updated abstract now specifies that the study utilized a comparative analytical cross-sectional design, conducted from April to July 2024 at the University Teaching Children’s Hospital and the HIV Pediatric Centre of Excellence in Lusaka, Zambia. We clarified that participants were children aged 2 to 15 years with no history of measles infection in the past six months, documented HIV status, and a minimum of two doses of measles vaccine. These changes make the abstract more directly representative of our study’s aim, providing a clear and specific summary of our approach, population, and inclusion criteria.

**Specification of Co-Infections in Tables**

**Comment:** For tables - can you specify co-infections further?

**Response:** We have added a footnote in the results table to clarify the classification of co-infections. In this study, co-infection refers to children who presented with additional conditions, such as tuberculosis, malaria, and diarrhea. Children with two or more infections were categorized as co-infected; otherwise, they were classified as not co-infected. This specification provides a clearer understanding of the types of co-infections considered and their impact on measles immunogenicity.

**Information on Past Measles Disease History**

**Comment:** Did you collect any info on measles disease status? Like past history of measles disease.

**Response:** information on past measles history was collected and we clarified in the methods section that children included in the study had no history of measles infection in the past six months, based on self-reported history and a review of medical records. This exclusion criterion was applied to focus on vaccine-induced immunity and minimize potential confounding from recent natural infection. No additional long-term history of measles infection was collected. We have added this detail to the methods for clarity.

**Impact of Breastfeeding on Measles Antibody Status**

**Comment:** Do you have any further comments or questions about how breastfeeding would be detrimental to measles antibody status?

**Response:** In our study, breastfeeding data were limited to a binary measure (breastfed or not), without further details on breastfeeding duration or exclusivity. Nonetheless, these initial findings align with evidence that breastfeeding may contribute to immune health, possibly through passive immunity transfer and other immunological benefits. We recognize that further research is needed to better understand the mechanisms through which breastfeeding may enhance vaccine efficacy, as well as to identify optimal breastfeeding practices. This discussion has been incorporated to underscore breastfeeding as a potentially beneficial factor in immunity retention.

**Algorithm Figure**

**Comment:** Comment on the participant recruitment algorithm.

**Response:** We have uploaded the participant recruitment algorithm on the Preflight Analysis and Conversion Engine (PACE) digital diagnostic tool to support transparency and reproducibility. This digital version enhances accessibility and provides a structured overview of our recruitment process, aiding readers in understanding the participant flow.

We hope these revisions align with the journal’s standards. We appreciate your guidance and look forward to your feedback.

Sincerely,

Dr. Priscilla Nkonde Gardner
