# Supplementary material for: Factors associated with measles vaccine immunogenicity in children at University Teaching Hospitals, Lusaka, Zambia
Source: PLOS Glob Public Health. 2025 Apr 23;5(4):e0003954. doi: 10.1371/journal.pgph.0003954 (PMC12017566; doi:10.1371/journal.pgph.0003954)
Supplement: Attachment — Submitted filename: Peer review comments from Mateus_Measles.docx [file pgph.0003954.s003.docx]

**Peer review comments from Mateus**

**Title:**

**Factors Associated with Measles Vaccine Immunogenicity in Children at University Teaching Hospitals, Lusaka, Zambia.**

**General comments**

1. Did collect assents from all the children included in the study? From what age the assent is valid in Zambia? Can a child of 2 years old provide a valid assent?
2. Please check the whole document and insert the references properly. It is not appropriate to insert reference after a dot (.) and neither inserting multiple references separately [(2), (3). Below is an example: “Immunization remains the most effective strategy for reducing morbidity and mortality from infectious diseases worldwide. However, the immune response to vaccines can vary considerably due to factors such as age, HIV status, and socio-demographic conditions. (2) (3).” This should be inserted like this: “… HIV status, and socio-demographic conditions (2, 3).
3. Check the tense used in writing in the whole document. There is a tension and mix of present and past tenses.

**Abstract**

“Residing in Lusaka was associated with lower immunity retention compared to children outside the province; …” => I am not sure this sentence is properly structured. Maybe say people who live in Lusaka showed lower immunity retention than those living outside …”.

**Introduction**

“The measles prevention initiative in Zambia seeks to reach 95 percent of children each year

through the routine immunization program”. => it is good to provide reference here.

The introduction is dominated by information about HIV positive and HIV negative children. It could better to have also information from the literature about breastfeeding, age in relation to measles immunity.

It would be better to have the aim of the study ang research questions at the end of the introduction.

**Methods**

“This comparative analytical cross-sectional study design was conducted from the 2nd of April to the 31st of July 2024”. => Can author write this section about study design properly. One sentence is not sufficient and it is not clear. Is it a retrospective study or prospective?

“The hospital recorded over 600 measles cases with about 20 deaths between 2022 and 2023

(19) ’’ => Can this sentence be part of the introduction (in the background) and explain this situation very well as part of the rationale for this study?

Data collected from April to July 2024, was this done prospectively? Was there a time and transport compensation for participants?

For inclusion and exclusion criteria, what about children who did not know their HIV status at the time of data collection? Were they tested by authors or just excluded from the study? How did authors approach participants for their enrollment in the study? Was there a community engagement programme?

“Children below the age of two years and above 15 years were and those with severe

immunodeficiency other than HIV, such as primary immunodeficiency disorders were excluded

from the study“. => what indicator did authors use for this?

“Univariable and multivariable logistic regression were employed to identify the

variables that predicted waning immunity in the mining community”. => I am not sure what this sentence means. Do you want to say “Univariate and multivariate analysis” ?

**Results**

It would be better to have a brief description of the results after the table.
