# Supplementary material for: Factors associated with measles vaccine immunogenicity in children at University Teaching Hospitals, Lusaka, Zambia
Source: PLOS Glob Public Health. 2025 Apr 23;5(4):e0003954. doi: 10.1371/journal.pgph.0003954 (PMC12017566; doi:10.1371/journal.pgph.0003954)
Supplement: Attachment — Submitted filename: Rebuttal Letter 8th January2025.docx [file pgph.0003954.s006.docx]

January 14, 2025

Dear Dr. Wagner,

**Re: Manuscript Resubmission PGPH-D-24-02312R1, "Factors Associated with Measles Vaccine Immunogenicity in Children at University Teaching Hospitals, Lusaka, Zambia"**

Thank you for providing the opportunity to revise and resubmit our manuscript. We appreciate the constructive comments and valuable feedback provided by the reviewers and yourself. Below, we provide a table summarizing the reviewers' comments, our responses, and the corresponding revisions made to the manuscript. We hope these changes meet the journal’s expectations.

**Reviewers Comments and Responses**

| **Reviewer** | **Comment** | **Response and Revision** |
| --- | --- | --- |
| Reviewer #3 | Inclusion and exclusion criteria: Mentioned only in the abstract. | Added detailed description of inclusion/exclusion criteria, including history of measles infection in the "Methods" section. |
| Reviewer #3 | Algorithm Figure: Lacked clarity on participant breakdown. | Reintroduced "S1 Fig" with participant breakdown and reasons for screen failures, discussed in "Methods." |
| Reviewer #3 | Tables 1, 2, 3 moved to supplementary material. | Restored Tables 1, 2, and 3 to the main manuscript for clarity and better flow. Revised references accordingly. |
| Reviewer #3 | Text corrections on age and breastfeeding. | Corrected text to: "Children aged 10-15 years had significantly lower odds of retaining immunity compared to those aged 2-4 years." Removed extraneous bracket before "AOR" and clarified breastfeeding-related results. |
| Reviewer #4 | Consolidate drafts; clarify text. | Ensured one clean version with tracked changes for resubmission. Revised unclear sentences throughout manuscript for clarity and flow. |
| Reviewer #4 | Quality of vaccine storage and cold chain. | Added discussion on the impact of vaccine storage quality in the "Introduction" section. |
| Reviewer #5 | Age categorization: Explore different age bins. | Conducted sensitivity analysis with alternative age groupings (e.g., 2-4, 5-7, 8-11, 12-15) and included results in "Results" and "Discussion." |
| Reviewer #5 | Confounders unclear. | Clarified in "Methods" section: Controlled for BMI, Education Status, Vaccination Timing, Coinfections, and Caregiver Educational Reviewer #5Status. Removed non-significant variables during stepwise regression. |
| Reviewer #5 | Booster dose recommendations. | Added quantitative evidence to support tailored booster programs based on sensitivity analysis results. |
| Reviewer #6 | Assent from children: Clarify age. | Specified in "Ethical Considerations" section: Assent obtained for children aged 7+, parental consent for younger children. |
| Reviewer #6 | References improperly formatted. | Reformatted references to adhere to journal guidelines. Grouped citations appropriately. |
| Reviewer #6 | Mixed tenses. | Revised manuscript for consistent past tense usage throughout. |
| Reviewer #7 | Justification for study missing. | Added justification at the end of the "Introduction," emphasizing public health relevance. |
| Reviewer #7 | Immunity vs. immunogenicity interchangeability. | Ensured consistent and accurate use of terms throughout manuscript. |
| Reviewer #7 | Measles immunity measurement. | Clarified methods for measuring immunity and definitions for "retained" vs. "waning" immunity in "Methods" section. |

**Summary of Changes:**

1. Addressed all reviewer comments with revisions reflected in the manuscript.
2. Improved clarity, consistency, and alignment with journal guidelines.
3. Included additional analyses and context to strengthen the manuscript.

Thank you once again for considering our manuscript. We look forward to your feedback and hope it meets the publication standards of PLOS Global Public Health.

Sincerely,

Priscilla Nkonde Gardner, MSc (On behalf of all co-authors)
