# Supplementary material for: Factors associated with measles vaccine immunogenicity in children at University Teaching Hospitals, Lusaka, Zambia
Source: PLOS Glob Public Health. 2025 Apr 23;5(4):e0003954. doi: 10.1371/journal.pgph.0003954 (PMC12017566; doi:10.1371/journal.pgph.0003954)
Supplement: Attachment — Submitted filename: Peer review comments from Mateus_Measles 2.docx [file pgph.0003954.s005.docx]

**Peer review comments from Mateus**

**Title:**

**Factors Associated with Measles Vaccine Immunogenicity in Children at University Teaching Hospitals, Lusaka, Zambia.**

**Abstract**

OK

**Introduction**

“Measles also results in severe complications such as pneumonia, encephalitis, and subacute sclerosing panencephalitis (SSPE), a progressive and fatal brain disorder that can develop years after the initial infection”. => This sentence needs reference.

“While these studies measure vaccine effectiveness in real-world settings using seropositivity as a marker, the findings underscore the need for careful monitoring of immunity levels across age groups and consideration of booster doses to sustain population-level immunity (8,9) for example, a study by Puthanakit in Thailand found that older children had significantly lower rates of measles seropositivity compared to younger children, highlighting the need for age appropriate vaccination strategies (10)”. => This sentence is too long and you can split it into 2.

For the third research question: “How do socio-demographic factors affect the retention of immunity in this population? “, is it possible to give some examples of socio-demographic factors? Do not assume that people know them. Aren’t age and breastfeeding part of these factors?

**Methods**

“To be included in the study, participants needed to be meet the following criteria: …” => correct grammar in this sentence.

“Data was collected from participants who visited the hospital between April and July using a complete enumeration”. => This is a repetition; it is already written above.

“Figure 1: Participant Algorithm …” => starting from this sentence, this paragraph should be part of inclusion and exclusion criteria or enrollment process. In the current version, it looks like being part of Ethics process.

**Results**

OK

**Discussion**

OK

**Conclusion**

Living in Lusaka was associated with waning immunity; however, … => put a dot after the word immunity and start However with a new sentence.
