# Supplementary material for: Factors associated with measles vaccine immunogenicity in children at University Teaching Hospitals, Lusaka, Zambia
Source: PLOS Glob Public Health. 2025 Apr 23;5(4):e0003954. doi: 10.1371/journal.pgph.0003954 (PMC12017566; doi:10.1371/journal.pgph.0003954)
Supplement: Attachment — Submitted filename: Rebuttal Letter 12th Feb 2025aaa.docx [file pgph.0003954.s008.docx]

12^th^ February 2025

**Re: Manuscript Resubmission PGPH-D-24-02312R2, "Factors Associated with Measles Vaccine Immunogenicity in Children at University Teaching Hospitals, Lusaka, Zambia"**

Dear Dr. Zimmerman and Reviewers,

Thank you for the opportunity to revise and resubmit our manuscript. We appreciate the constructive feedback and have carefully addressed all the points raised by the reviewers. Below is our detailed response to each comment:

| **Reviewer** | **Comment** | **Response** |
| --- | --- | --- |
| **Reviewer #1** | The conclusion should contain only the main findings in relation to the objective of the study. | We have revised the conclusion section to focus on the main findings of the study in direct relation to the objectives. |
| **Reviewer #1** | The ELISA kit should be evaluated with the gold standard. Otherwise, this will hamper the outcomes of the study. | We acknowledge this concern and have included a statement in the Methods section describing the performance of the ELISA kit in comparison to standard reference assays. |
| **Reviewer #5** | Please make sure to check for typos. For example, the sensitivity analysis section states: "Children aged 10-15 years had significantly lower odds of retaining immunity compared to younger children (AOR: 0.159; 95% CI: 1.049-1.631, p=0.002)." The age category should likely be "12-15 years" and the confidence interval does not match the odds ratio. | Thank you for this comment. We have corrected the age category to "12-15 years" and verified the confidence interval to ensure consistency with the odds ratio. |
| **Reviewer #6** | Suggested title change to: "Factors Associated with Measles Vaccine Immunogenicity in Children at University Teaching Hospitals, Lusaka, Zambia." | We have updated the title as suggested. |
| **Reviewer #6** | "Measles also results in severe complications such as pneumonia, encephalitis, and subacute sclerosing panencephalitis (SSPE), a progressive and fatal brain disorder that can develop years after the initial infection." Needs a reference. | We have added a relevant reference supporting this statement. |
| **Reviewer #6** | "While these studies measure vaccine effectiveness in real-world settings using seropositivity as a marker, the findings underscore the need for careful monitoring of immunity levels across age groups and consideration of booster doses to sustain population-level immunity (8,9) for example, a study by Puthanakit in Thailand found that older children had significantly lower rates of measles seropositivity compared to younger children, highlighting the need for age-appropriate vaccination strategies (10)." This sentence is too long; consider splitting it. | We have split this sentence into two for clarity. |
| **Reviewer #6** | For the third research question, "How do socio-demographic factors affect the retention of immunity in this population?" Can you give some examples of socio-demographic factors? | We have clarified this in the introduction by providing examples such as "parental education level, household income, and urban versus rural residence." |
| **Reviewer #6** | "To be included in the study, participants needed to be meet the following criteria: ..." Correct grammar in this sentence. | The sentence has been corrected. |
| **Reviewer #6** | "Data was collected from participants who visited the hospital between April and July using a complete enumeration." This is a repetition. | We have removed the redundant sentence to improve clarity. |

- **Reference List:** We have reviewed and confirmed that all references are complete, accurate, and up-to-date. No retracted references have been cited.
- **Data Availability Statement:** We have ensured that all data supporting the findings are available as per PLOS policies.
- **English Language and Grammar:** We have proofread the entire manuscript to correct typographical and grammatical errors.

We appreciate the time and effort invested by the reviewers and editors in improving our manuscript. We believe these revisions strengthen our study, and we hope it is now suitable for publication in PLOS Global Public Health.

Thank you for your consideration. We look forward to your feedback.

**Sincerely,**
Dr. Priscilla Nkonde Gardner
Corresponding Author
