# Supplementary material for: Factors associated with measles vaccine immunogenicity in children at University Teaching Hospitals, Lusaka, Zambia
Source: PLOS Glob Public Health. 2025 Apr 23;5(4):e0003954. doi: 10.1371/journal.pgph.0003954 (PMC12017566; doi:10.1371/journal.pgph.0003954)
Supplement: Attachment — Submitted filename: Peer review comments from Mateus_Measles 3.docx [file pgph.0003954.s007.docx]

**Peer review comments from Mateus**

**Title:**

**Factors Associated with Measles Vaccine Immunogenicity in Children at University Teaching Hospitals, Lusaka, Zambia.**

**Abstract**

OK

**Introduction**

The sentence “Insight into these factors were important inform policy and interventions on measles vaccination programs in Zambia” should read “Insight into these factors were important to inform policy and interventions on measles vaccination programs in Zambia”.

**Methods**

The sentence “Participants who were included in this study meet the following criteria:…” should read “Participants who were included in this study met the following criteria:…”

**Results**

OK

**Discussion**

OK

**Conclusion**

OK
