# Supplementary material for: Factors associated with measles vaccine immunogenicity in children at University Teaching Hospitals, Lusaka, Zambia
Source: PLOS Glob Public Health. 2025 Apr 23;5(4):e0003954. doi: 10.1371/journal.pgph.0003954 (PMC12017566; doi:10.1371/journal.pgph.0003954)
Supplement: Attachment — Submitted filename: Rebuttal Letter 10thmarch.docx [file pgph.0003954.s009.docx]

**Rebuttal Letter**

Priscilla Nkonde Gardner

University of Zambia
10^th^ March 2025

The Editor

PLOS Global Public Health

Subject: Response to Reviewer and Editor Comments for Manuscript PGPH-D-24-02312R3

Dear Dr. Wagner,

Thank you for your detailed review and for providing us the opportunity to revise and improve our manuscript titled "Predictors of Measles Vaccine Immunogenicity in Vaccinated Children at University Teaching Hospitals, Lusaka, Zambia." We appreciate the constructive feedback and have made the necessary changes as requested. Below, we provide a point-by-point response to the editor and reviewer comments.

**Editor’s Comments:**

1. **Abstract:** The sentence regarding the exclusion of variables (carer education level, co-infections, and child education status) and the explanation of the alpha level were deemed unnecessary.
   - *Response:* We have removed these sentences and instead provided a summary of key descriptive results, including the number of children with and without HIV and their measles seropositivity.
2. **Introduction:** The paragraph on vaccine storage and cold chain should be moved to the discussion.
   - *Response:* We have moved this section to the discussion as suggested, to better align with the study’s focus on immunogenicity differences in HIV+ and HIV- individuals.

**Reviewer #6 Comments:**

1. **Introduction:** The sentence "Insight into these factors were important inform policy and interventions on measles vaccination programs in Zambia" needs grammatical correction.
   - *Response:* We have revised it to "Insight into these factors is important to inform policy and interventions on measles vaccination programs in Zambia."
2. **Methods:** The sentence "Participants who were included in this study meet the following criteria:..." needs grammatical correction.
   - *Response:* We have revised it to "Participants who were included in this study met the following criteria:..."

**Conclusion:**

We appreciate the constructive feedback and believe that these changes have strengthened our manuscript. We hope that the revised version now meets the publication criteria of PLOS Global Public Health. Thank you for your time and consideration, and we look forward to your positive response.

Sincerely,
Priscilla Nkonde Gardner
